# Rainy-Day Thoughts

**Published:** 1903-04

**Authors:** G. Chisholm

**Affiliations:** Birmingham, Ala.


### Domestic Correspondence.

### RAINY-DAY THOUGHTS.

The article in the November issue of the International Dental Journal, headed "Putrescent Pulps: A Criticism," is interesting, not because it throws any light upon the treatment of teeth in that condition, but because it simply shows how little some men, engaged in the practice of dentistry, know about the pathological condition of teeth which daily come before them for treatment.

Of course, these young graduates all come from prominent colleges. "So are they all," prominent colleges of the highest standing. And the faculties are composed of "all honorable men."

Yet there are honorable men who do not know how to shoe a horse, conduct a case in court, treat a diseased tooth, or set a broken limb. The fault, therefore, is not always entirely that of the college or graduate, but largely due to both.

The chair of pathology sometimes makes a heavier impression on the rostrum than it does upon the mind of the student, and yet it may not be a very heavy-weight chair. The student may be thick-headed and lacking in his appreciation of the fact that there is more in dentistry than the mere art of mechanics. But criticism is easy, and there is no end to it. What we all want to be able to do is, first, to be able to recognize the troubles which come before us, and when we do, we should be able to make a correct diagnosis; then a treatment will suggest itself to fit each case, and there need be no guesswork about it, and precautions regarding septic conditions and septic poison should always be considered, not for the purpose of alarming the patient, making a big charge, or trying to impress the infallible "ego" of some men, but because of the comfort to your own mind and conscience in knowing that you have done the best you could for your suffering patient, and resting assured that it will be fully appreciated and you will receive a fair reward for your services if you regard them worth anything; and if you do not, who will?

In conclusion, I will offer a few remarks on the treatment of putrescent conditions in root-canals. Where there is soreness and pus, there is nothing in my hands which gives more ready and reliable relief than tincture of iodine used freely in the cavity and root-canal. Then use as a cleansing agent pyrozone. After washing the cavity thoroughly with this, sterilize the cavity with either sodium dioxide or ordinary hydrate of soda, being careful not to allow the sterilizing agents to come in contact with the gum or pass through the ends of the root-canal. I usually apply it by dipping a small nerve instrument into the preparation and passing it into the canals as far as possible; then wash out with alcohol, remove the alcohol with chloroform, and fill with chloropercha. By this method of treatment my experience is that immediate root-filling is possible. However, the amount of soreness in the peridental membrane must be considered, and if the inflammation is excessive, I prefer simply the tincture of iodine treatment, at the first sitting, completing the operation at the second.

G. Chisholm.

Birmingham, Ala.

